# Round-robin testing for LMO2 and MYC as immunohistochemical markers to screen *MYC* rearrangements in aggressive large B-cell lymphoma

**DOI:** 10.1007/s00428-023-03584-9

**Published:** 2023-06-27

**Authors:** Natalia Papaleo, Fina Climent, Gustavo Tapia, Luis Luizaga, Juan Azcarate, Jan Bosch-Schips, Ana M. Muñoz-Marmol, Marta Salido, Carmen Lome-Maldonado, Ivonne Vazquez, Luis Colomo

**Affiliations:** 1 Department of Pathology, Parc Taulí Hospital Universitari, Institut d’Investigació i Innovació Parc Taulí (I3PT-CERCA), Barcelona, Spain; 2 Universitat Autonoma de Barcelona, Barcelona, Spain; 3 Universitat Pompeu Fabra, Barcelona, Spain; 4 Department of Pathology, Hospital Universitari de Bellvitge, L’Hospitalet de Llobregat, Barcelona, Spain; 5 Department of Pathology, Hospital Universitari Germans Trias I Pujol, Badalona, Barcelona, Spain; 6 Department of Pathology, Hospital Mutua Terrassa, Terrassa, Barcelona, Spain; 7 Department of Pathology, Hospital del Mar, Institute Hospital del Mar d’Investigacions Mediques (IMIM), Barcelona, Spain

**Keywords:** Lymphomas, Large B-cell lymphoma, MYC, LMO2, FISH

## Abstract

Aggressive large B-cell lymphomas (aLBCL) include a heterogeneous group of lymphomas with diverse biological features. One of the approaches to the diagnosis of aLBCL is based on the identification of *MYC* rearrangements (*MYC*-R), in addition to *BCL2* and *BCL6* rearrangements by genetic techniques, mainly fluorescent in situ hybridization (FISH). Because of the low incidence of *MYC*-R, the identification of useful immunohistochemistry markers to select cases for *MYC* FISH testing may be useful in daily practice. In a previous work, we identified a strong association between the profile CD10 positive/LMO2 negative expression and the presence of *MYC*-R in aLBCL and obtained good intralaboratory reproducibility. In this study, we wanted to evaluate external reproducibility. To evaluate whether LMO2 can be a reproducible marker between observers 50 aLBCL cases were circulated among 7 hematopathologists of 5 hospitals. Fleiss’ kappa index for LMO2 and MYC were 0.87 and 0.70, respectively, indicating high agreement between observers. In addition, during 2021–2022, the enrolled centers included LMO2 in their diagnostic panels to evaluate prospectively the utility of the marker, and 213 cases were analyzed. Comparing LMO2 with MYC, the group of CD10 positive cases showed higher specificity (86% vs 79%), positive predictive value (66% vs 58%), likelihood positive value (5.47 vs 3.78), and accuracy (83% vs 79%), whereas the negative predictive values remained similar (90% vs 91%). These findings place LMO2 as a useful and reproducible marker to screen *MYC*-R in aLBCL.

**Supplementary Information:**

The online version contains supplementary material available at 10.1007/s00428-023-03584-9.

## Introduction

Aggressive large B-cell lymphomas (aLBCL), including transformed B-cell lymphomas from low-grade non-Hodgkin lymphomas and Burkitt lymphoma (BL), are the most common lymphomas causing tissue involvement in western countries [1]. Although chronic lymphocytic leukemia/small lymphocytic lymphoma has higher incidence than aLBCL, the disease is largely limited to the peripheral blood, and the diagnostic approach of the disease is not based on tissue examination [2]. It is known that the status of *MYC* gene is prognostically relevant in aLBCL.

The rearrangements involving *MYC* (*MYC*-R) are a defining genetic alteration of high-grade B-cell lymphomas (HGBL) carrying *BCL2* and/or *BCL6* rearrangements, as well as Burkitt lymphoma (BL) [3, 4]. Furthermore, *MYC*-R have a prevalence of 5–15% in diffuse large B-cell lymphoma, not otherwise specified (DLBCL-NOS), which is the most common subtype of aLBCL. This lymphoma represents a morphologically, genetically, and clinically heterogeneous entity and the detection of *MYC-*R associates with a poorer outcome after standard chemoimmunotherapy, as HGBL carrying *MYC* and *BCL2* rearrangements [3, 5, 6]. In addition, 10 to 26% transformed DLBCL (tDLBCL) carry *MYC*-R [7, 8]. Thus, all these data indicate the need of the identification of *MYC* status in aLBCL.

There has been an extraordinary increase in the knowledge of hematological neoplasms since the publication of the unified REAL classification [9, 10]. New genetic tools, gene expression profiling (GEP), and next-generation sequencing (NGS) have expanded the understanding of the biology of aLBCL. Progress in the understanding of aLBCL points to a more refined classification including the combination of molecular and genetic data that ideally should also include suitable information obtained from morphology and immunohistochemistry (IHC) [3, 11]. However, the current strategy to diagnose aLBCL in most laboratories relies on the use of IHC combined with cytogenetics, where available. Genetic testing is mandatory for the classification of aLBCL [3, 4, 12]. Since the overall incidence of *MYC*-R in LBCL is low, and cytogenetics is not available elsewhere, it is necessary to identify useful markers to screen *MYC*-R in routine practice. In previous studies, we observed the utility of the association between LMO2 loss of expression by IHC with the presence of *MYC*-R in aLBCL [7, 13, 14].

LMO2 is a cysteine-rich protein which is a critical regulator of hematopoiesis, initially described as a recurrent chromosomal translocation partner of the *TCR* genes associated with T-cell acute lymphoblastic leukemia [15]. GEP studies included *LMO2* among the genes defining the GCB-like profile signature [16, 17]. It is currently known that LMO2 is expressed in aLBCL and that the immunohistochemical expression of LMO2 has an impact in the survival of patients treated with immunochemotherapy [7, 18]. The favorable prognosis has been related to mechanisms of genomic instability associated with DNA damage [19, 20].

Our previous studies showing the utility of LMO2 as a marker to identify *MYC*-R included two independent series of 330 and 365 samples, shared methods, and obtained similar results, unveiling intralaboratory reproducibility [7, 13]. Two studies published later including 90 and 180 aLBCL, respectively, showed similar results to ours [21, 22]. In the present study, we aimed to evaluate the interobserver and interlaboratory reproducibility for LMO2 and MYC detected by IHC in aLBCL. We proceeded in two phases. In the first phase, 50 aLBCL cases from one center, collected retrospectively, were circulated to evaluate the interobserver concordance of IHC. The second phase of the study was conducted prospectively, aiming to evaluate the performance of each laboratory. Thus, each enrolled hospital collected their in-house aLBCL, adding LMO2 antibody to their diagnostic panel. The results of the immunohistochemical panel were correlated with *MYC* FISH results obtained from each laboratory. At the same time, as we were collecting such prospective data, we also pretended to identify the incidence of *MYC*-R in the centers involved in the study.

## Material and methods

To analyze interobserver reproducibility we performed a round-robin test. Fifty aLBCL diagnosed between 2016 and 2021 were selected from the files of the Pathology laboratory of the Hospital del Mar, Barcelona, based on available material. All cases were diagnosed according to the 4th revised WHO classification [12]. Primary mediastinal large B-cell lymphoma, primary central nervous system lymphoma and HIV-associated lymphomas were excluded. The series included whole tissue sections of 28 excisional biopsies (EB) and 22 core needle biopsies (CNB). Each case comprised a set of slides including hematoxylin and eosin, CD10 (clone SP67), BCL6 (clone GI191E-A8), MUM-1/IRF4 (clone MRQ-43), BCL2 (clone 124), LMO2 (clone 1A9-1), and MYC (clone Y69), all from Ventana, Roche, Tucson, AZ, USA. The immunohistochemical studies were performed, as previously described [13]. During 2020–2021, all cases were circulated and evaluated by 7 hematopathologists (FC, GT, IV, CL-M, LL, NP, and LC) from 5 tertiary hospitals located in the health area of Barcelona, Spain (Hospital de Bellvitge, center 1; Hospital Germans Trias i Pujol, center 2; Hospital del Mar, center 3; Hospital Parc Tauli, center 4; Hospital Mutua Terrassa, center 5), in 2 to 4 individual sessions. The evaluation and assessment for all the antibodies were the same, as previously described [13]. The cutoff used for CD10, BCL6, MUM1/IRF4, and LMO2 was 30%, and for MYC and BCL2 was 40% and 50%, respectively. Lymphoma diagnoses and FISH results of *MYC*, *BCL2*, and *BCL6* were blinded for all observers. Split probes for *MYC* and *BCL6* and dual fusion probes for *BCL2/IGH* and *MYC/IGH* were all provided by Vysis, Abbott Molecular, and Des Plainescity, IL, USA. FISH was performed and evaluated, as described following the criteria of Ventura [23].

The second phase of the study corresponded to the interlaboratory reproducibility phase. A prospective study was performed from January 2021 to June 2022 by each center. Samples of daily practice with a diagnostic suspicion of aLBCL as per the 4th revised WHO classification were stained with CD10, BCL2, BCL6, MUM1/IRF4, and MYC, according to the protocols of each laboratory. Same entities as in phase one were excluded. Clones and sources are described in supplementary Table 1. LMO2 was included in the immunohistochemical panel for the diagnostic workout in all cases. *MYC*, *BCL2*, and *BCL6* FISH probes were performed and interpreted according to the protocols and probes of each laboratory (supplementary Table 1). Each center was asked to fill in an Excel template including blinded ID number, patient data (age, sex, and relevant medical history), IHC and *MYC* FISH results, and diagnosis. This series include patients diagnosed and treated in each institution corresponding to their health areas of influence. Some differences in terms of healthcare services between the centers exist: centers 1 and 2 receive patients needing complex treatments such as allogenic transplant and CAR-T cell therapy, and their health area covers a population of 1.3 million and 800.000 inhabitants, respectively. Centers 3 to 5 cover similar health areas in terms of the number of population that includes approximately 400,000 inhabitants. Complex treatments are referred to other centers, different to centers 1 and 2. Center 3, in addition, centralize cases for diagnosis-genetic testing. The approach to FISH testing was also variable, since centers 2 and 3 used *MYC/IGH* probes to determine the partner of MYC*-R* cases, and center 2 only tested *BCL2* and *BCL6* FISH for *MYC*-R cases. Patient samples were collected in accordance with the Declaration of Helsinki and approved by ethics committee.

In the present study, we decided to keep the nomenclature of the revised 4th edition of the WHO classification, as it was developed between January 2020 and June 2022. We have only modified Burkitt-like lymphoma with 11q aberration included in the revised 4th edition of the WHO classification and used the mixed term high grade/large B-cell lymphoma with 11q aberrations (HGBL/LBCL-11q), as handled in the 21st EAHP-SH meeting in Florence, 2022.

### Statistical analysis

To quantify the agreement between observers in the phase 1 of the study we used the Fleiss’ kappa index. *χ*^2^ test, unpaired *t* tests, or non-parametric tests, were used when necessary.

For the second phase, accuracy, sensitivity, specificity, and positive/negative predictive ratios were calculated for MYC and LMO2. Likelihood positive and negative ratios were calculated to evaluate the diagnostic accuracy of the results obtained. *P* values < 0.05 were considered statistically significant for all tests.

Data were analyzed using Microsoft Excel 2019 (Microsoft Corporation, Redmond, WA, USA) and the v28.0.1.0 of the software package IBM SPSS Statistics (Armonk, NY, USA).

## Results

### Interobserver reproducibility

In this phase, we analyzed 50 cases including 2 (4%) HGBCL-NOS, 8 (16%) high-grade B-cell lymphoma with *MYC* and *BLC2/BCL6* rearrangement (HGBCL with *MYC-BLC2/BCL6* R), 31 (62%) DLBCL-NOS, 7 (14%) tDLBCL (6 from follicular lymphoma-FL; 1 from marginal zone lymphoma, MZL), 1 (2%) BL and 1 (2%) HGBCL/LBCL-11q. The overall incidence of *MYC*-R in the series was 28% (14/50 cases): 1 HGBCL-NOS, 8 HGBCL with *MYC-BLC2/BCL6* R, 4 DLBCL-NOS and 1 BL. Any tDLBCL included in the series presented *MYC*-R.

The patients were 32 males and 18 females, with a median age of 62 years (range 34–91). Thirty-three (66%) cases were nodal and 17 (34%) extranodal. The samples were obtained by excisional biopsies (EB) in 28 (56%) cases and 22 (44%) were core needle biopsies (CNB).

**Fig. 1:**
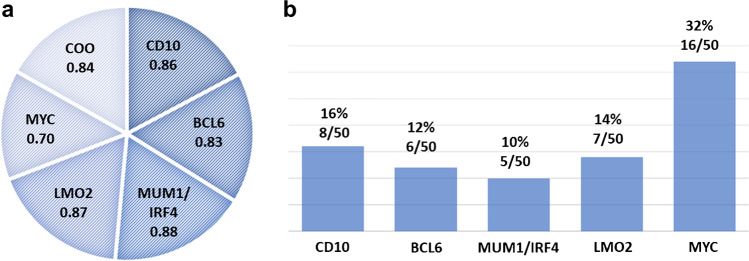
Fleiss’ kappa index by each IHC marker and COO by Hans algorithm, including all cases (*N* = 50) (**a**). Frequency of discrepancies between the observers for each marker (**b**)

We first analyzed how was the concordance of CD10, BCL6, and MUM1/IRF4 to assess the COO of all cases included in this series and in DLBCL-NOS, to compare our results with the previously published by other groups. Fleiss’ Kappa index for COO concordance was 0.84, considering all cases included in the series, and 0.77 for DLBCL-NOS only (*P* < 0.001 each). The concordance analysis for the markers using the same approach (total cases and DLBCL-NOS only) was CD10, 0.86/0.79; BCL6, 0.83/0.80; and MUM1/IRF4, 0.88/0.83. For LMO2 and MYC the results were: LMO2, 0.87/0.89; and MYC: 0.70/0.64 (*P* < 0.001 for each marker). CD10, BCL6, and MUM1/IRF4 and LMO2 staining obtained high agreement values, whereas the lowest concordance rate was obtained for MYC staining, particularly when the COO analysis was considered (Fig. 1).

**Fig. 2:**
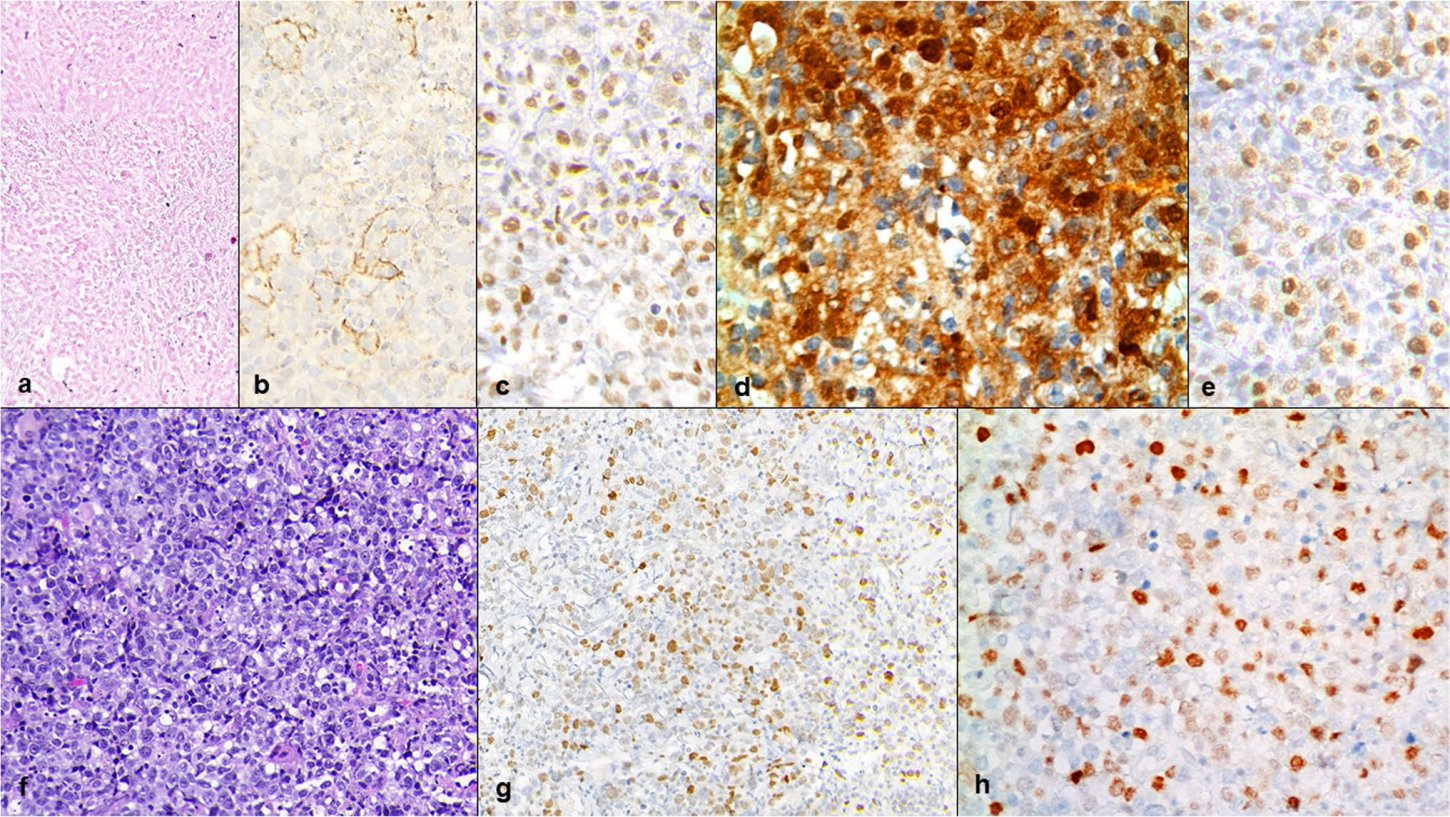
HGBCL with *MYC*-R and *BCL6*-R, diagnosed by CNB of a retroperitoneal lymph node with necrotic areas (**a**), CD10 negative (**b**), BCL6 positive (**c**), LMO2 negative with intermediate discrepancies (5/2) (**d**), and MYC positive with no discrepancies (**e**). DLBCL-NOS, CD10 negative, and LMO2 negative with no *MYC*-R diagnosed in EB (**f**) and with major discrepancies (4/3) in MYC staining (**g**, **h**)

Table 1 summarizes the causes of disagreement in the IHC evaluation between the observers. We classified discrepancies as major when 3 observers disagreed; intermediate when 2 observers disagreed; and minor when only 1 observer disagreed. The discrepancies for LMO2 occurred in 7/50 (14%) cases and were 2 major, 2 intermediate, and 3 minor. The causes of LMO2 discrepancies were primarily attributed to the differences in the interpretation of the cutoff for LMO2 between observers. Interestingly, 6 of 7 cases were CD10 negative and, as published, the level of expression of LMO2 is variable in such cases [13, 24]. Three of 7 cases were non-GCB-like according to Hans’ algorithm. Among the 4 GCB-like, there were 2 DLBCL-NOS, 1 HGBCL with *MYC-BLC2/BCL6*, and 1 HGBCL-NOS. Additional variability associated with major and intermediate discrepancies were attributed to the size of the samples and the quality of the tissue in 3 cases. All were CNB, 2 with necrotic areas, and 1 with areas of bad fixation. Minor discrepancies occurred in CD10 negative cases (1 GCB and 2 non-GCB-like) (Fig. 2a–e).

**Table 1:** Causes of disagreement between LMO2 and MYC in the interobserver reproducibility study

Discrepancies	LMO2	MYC
Frequency	7/50 (14%)	16/50 (32%)
Type*	2 Major/2 intermediate/3 Minor	6 Major/5 intermediate/5 minor
Sample size	3/7 (43%) discrepant cases were CNB	11/16 (69%) discrepant cases were CNB
Sample artifacts	3/7 (43%)	3/16 (19%)
Fixation	- 2 CNB	- 2 EB
Other	- 1 CNB with important necrosis	- 1 CNB with crush artifact
Differences in cutoff interpretation	6/7 (88%) discrepant cases were CD10-negative, 4 GCB-like	13/16 (81%) cases had MYC expression included in the quartile 25 to 50%

*Type: major, indicates 3-observer discordance; intermediate, indicates 2; minor, only 1 observer disagreed; CNB: core needle biopsy; EB: excisional biopsy

Discrepancies for MYC occurred in 16/50 (32%) cases and were 6 major, 5 intermediate, and 5 minor. In 13 of 16 (81%) discrepant samples, MYC expression varied between 25 and 50%, and the observers agreed with that it was difficult to determine whether the tumor cells were over or not the cutoff defined for MYC (Fig. 2f–h). The size of the sample was also important, since 11/16 (69%) discrepancies occurred in CNB. Additional discrepancies were attributed to the quality of the samples in 3 cases. Two EB had fixation issues that caused irregular staining of MYC, and 1 CNB had a crush artifact. Only one case, an EB CD10 negative and non-GCB-like, presented simultaneous discrepancies for LMO2 and MYC that were minor and intermediate, respectively.

### Interlaboratory reproducibility

The second phase of the study included 213 cases which were collected during a period of 18 months. Briefly, centers 1 to 5 included 55, 35, 69, 36, and 18 cases, respectively.

Table 2 shows the results of all aLBCL included. Overall, the whole series comprised 4 HGBCL-NOS, 19 HGBCL with *MYC-BLC2/BCL6* R, 150 DLBCL-NOS, 33 tDLBCL (28 transformed FL and 5 transformed MZL), and 7 BL. The median age of the patients was 68 years (range 19–92 years). About 141 cases were nodal and 72 extranodal. After excluding BL, and following the Hans algorithm, 118 (57%) cases were GCB-like (69 DLBCL-NOS, 4 HGBCL-NOS, 18 HGBCL with *MYC-BLC2/BCL6* R, and 27 tDLBCL), and 88 (43%) cases were non-GCB-like (81 DLBCL-NOS, 1 HGBCL with *MYC-BLC2/BCL6* R, and 6 tDLBCL). Considering the whole series, 34/213 cases harbored *MYC*-R, with an overall incidence of 16%: 7 BL, 7 DLBCL-NOS, 19 HGBCL with *MYC-BLC2/BCL6* R, and 1 tDLBCL. CD10 was expressed in 101 cases (47%), LMO2 in 132 cases (62%), and MYC in 78 cases (37%).

**Table 2:** Diagnosis and distribution of COO, LMO2 and MYC expression, and FISH rearrangements of the whole series, including 213 cases

	Center 1 *N* = 55	Center 2 *N* = 35	Center 3 *N* = 69	Center 4 *N* = 36	Center 5 *N* = 18
Diagnosis					
DLBCL-NOS	34 (62%)	24 (69%)	46 (67%)	28 (78%)	18 (100%)
tDLBCL	6 (11%)	4 (11%)	17 (25%)	6 (17%)	0
HGBCL with *MYC-BLC2/BCL6* R	10 (18%)	5 (14%)	3 (4%)	1 (3%)	0
HGBCL-NOS	2 (4%)	1 (3%)	0	1 (3%)	0
BL	3 (5%)	1 (3%)	3 (4%)	0	0
Immunohistochemistry					
CD10	28 (51%)	15 (43%)	35 (51%)	13 (36%)	10 (56%)
BCL6	48 (87%)	28 (80%)	65 (64%)	32 (89%)	18 (100%)
BCL2	34 (62%)	21 (60%)	52 (75%)	29 (72%)	18 (100%)
MUM-1/IRF4	26 (47%)	22 (63%)	41 (59%)	25 (64%)	11 (61%)
LMO2	38 (69%)	12 (34%)	43 (62%)	24 (67%)	15 (83%)
MYC	34 (62%)	12 (34%)	14 (20%)	10 (28%)	8 (44%)
FISH					
*MYC-R*	14 (25%)	9 (26%)	9 (13%)	2 (6%)	0
*BCL2*-R	18 (33%)	6 (67%)*	18 (26%)	11 (31%)	7 (39%)
*BCL6*-R	20 (36%)	5 (55%)*	24 (35%)	9 (25%)	8 (44%)
COO					
GCB-like	36 (69%)	17 (50%)	37 (56%)	17 (47%)	11 (61%)
Non-GCB-like	16 (31%)	17 (50%)	29 (44%)	19 (53%)	7 (39%)

*Results related to 9 *MYC*-R cases, since center 2 tests *BCL2* and *BCL6* when *MYC* is rearranged

The statistic measures of the performance of LMO2 and MYC compared with the presence of *MYC*-R as the gold standard of all cases included in the series and CD10 positive cases are shown in Table 3. Center 5 is not included, since no *MYC*-R were detected. Supplementary Tables 2 and 3 show the results by center, the Hans algorithm, and double expression of BCL2 and MYC proteins. As expected, the results obtained for LMO2 in CD10 positive cases ameliorated the results of the whole series, except for the negative predictive value (NPV). Comparing LMO2 with MYC, the group of CD10 positive cases showed higher values for the specificity (86% vs 79%), positive predictive value (66% vs 58%), likelihood positive value (5.47 vs 3.78), and accuracy (83% vs 79%), whereas the NPV remained similar (90% vs 91%). Remarkably, taking into account the variability of sources and approaches used for the diagnosis of *MYC*-R in each laboratory, the overall results were similar to those obtained in our two previous studies (Table 3). Specially, high similar values for the specificity and NPV were obtained in the three studies.

**Table 3:** Statistic measures of LMO2 and MYC protein expression using *MYC*-R as the gold standard

	All cases LMO2 *N* = 213	All cases MYC *N* = 210	CD10+ cohort LMO2 *N* = 101	CD10+ cohort MYC *N* = 100	CD10+ cohort LMO2 Ref #13 *N* = 98	CD10+ cohort LMO2 Ref #7 *N* = 175
Sensitivity	71%	76%	75%	78%	87%	79%
Specificity	68%	71%	86%	79%	87%	89%
PPV	29%	32%	66%	58%	74%	81%
NPV	92%	94%	90%	91%	94%	88%
Positive LR	2.21	2.57	5.47	3.78	6.69	7.19
Negative LR	0.43	0.34	0.29	0.28	0.14	0.24
Accuracy	68%	71%	83%	79%	87%	85%

*PPV* positive predictive value; *NPV* negative predictive value; positive *LR* positive likelihood ratio; negative *LR* negative likelihood ratio

Overall, we identified 16 dissociated cases that were as follows: 7 cases carrying *MYC*-R showed double positive expression of CD10 and LMO2 (CD10+/LMO2+/*MYC*-R); and 9 cases with CD10+/LMO2- phenotype in which we did not identify *MYC*-R (CD10+/LMO2-/no-*MYC*-R). Among the 7 CD10+/LMO2+/*MYC*-R cases, 4 had MYC protein expression over 40%. On the contrary, 6 out of 9 cases showing CD10+/LMO2-/no-*MYC*-R profile, had expression of MYC by IHC below 40%. Finally, the incidence of *MYC*-R varied among centers (center 1: 25; center 2: 26, center 3: 13; center 4: 6; and center 5: 0%). As centers 1 to 3 receive external patients and consultation cases, we wanted to clarify the real incidence of *MYC*-R in our series. After excluding the referred cases, centers 1 to 3 had an incidence for *MYC*-R of 23%, 19%, and 7%, respectively.

## Discussion

In this study, we aimed to evaluate the clinical reproducibility of LMO2 identified by IHC in aLBCL. To evaluate the interobserver reproducibility, we used a similar strategy to other studies [25, 26] and selected a set of cases that were independently evaluated by 7 hematopathologists. We realized that we agreed in the interpretation of the markers included in the Hans algorithm, as other authors described previously [25–28], and these results encouraged us to analyze LMO2 and MYC.

We observed fewer discrepancies for LMO2 than MYC and attributed primarily the disagreement to the interpretation of the cutoff used. For LMO2, most discrepancies occurred in CD10 negative cases, and these are the aLBCL showing higher variability of LMO2 expression. In our previous studies, we observed that LMO2 protein expression was very high in CD10 positive cases and mostly negative in *MYC*-R cases, showing very low variability. However, CD10 negative and non-GCB-like tumors showed more fluctuating expression of LMO2, ranging from 10 to 40% [7, 13]. Such variability was already observed in GEP studies, where ABC and unclassifiable aLBCL had high levels of mRNA LMO2, particularly among tumors with no *MYC*-R. When *MYC*-R where present in such cases, LMO2 was low in unclassified but higher values persisted in the ABC subtype [13, 24].

MYC disagreement occurred mostly in CNB with values of MYC expression ranging from 25 to 50%. Our results are similar to those obtained in the study of Mahmoud [29] that analyzed two independent sets of cases and evaluated whole tissue slides, including a total number of 35 aLBCL (5 BL and 30 DLBCL). In this study, the authors obtained an overall Kappa score of 0.68 and attributed such moderate concordance to the inherent heterogeneity of MYC expression in DLBCL. They concluded the need to be cautious when interpreting cases with MYC staining close to 40%. Moreover, the authors showed that the preselection of fields of 1 mm, as used in TMA concordance studies, improved the agreement between observers, but did not eliminate discrepancy at all. In summary, our results indicate higher agreement between observers for LMO2, compared to MYC.

In the second phase of the study, we wanted to know how useful was the inclusion of LMO2 in the immunohistochemical panels used for the work up of aLBCL. Then, all centers used the same clone and conditions for LMO2, but did not add changes to their protocols routinely used for the additional markers. Notably, analyzing the total number of cases diagnosed by the five centers, we obtained similar results to our previous studies. In comparison with the results of such series[7, 13], we observed a slight decrease in the sensitivity, PPV, and positive likelihood ratio in the multicenter study, values concerning the ability to identify the association between LMO2 loss and *MYC*-R presence. We realized that the approaches to the detection of *MYC*-R were variable among centers in terms of sources of the probes used, usage of the probes, and interpreters of the FISH technique. It is known that the approach to the diagnosis of *MYC*-R may influence the ability to detect such genetic alteration [30]. Then, since the methods to study *MYC*-R may be quite heterogeneous in the real world, the identification of additional markers should help to evaluate the cytogenetic results after FISH testing. Ancillary markers may also help to suspect the presence of *MYC* cryptic insertions that may occur in aLBCL and decrease the number of false negative cases carrying *MYC*-R not detected by FISH [31, 32]. In the present study, we have also compared the utility of classifiers such as the Hans algorithm and double expression of MYC and BCL2 proteins to detect *MYC*-R. The results are shown in supplementary Table 3 and do not improve the CD10/LMO2 approach.

Our cohort of CD10 positive cases lacking LMO2 expression predicted the presence of *MYC*-R with high levels of specificity, accuracy, positive and negative predictive values, and good positive and negative likelihood ratios. We decided to analyze the multicenter results as a unique series assuming the variability of the diagnostic approaches to avoid the bias induced in the screening tests when the number of cases studied is low. With this approach, the specificity, NPV and accuracy were 86%, 90%, and 83%, respectively. When we analyzed the same parameters per center, we observed higher variability due to the lower number of cases included in each center. However, considering the individual results, one center obtained a value of NPV around 80%, one around 90%, and two obtained values of 100%. By using the profile CD10+/LMO2-, it is desirable to obtain very high NPV to avoid false negative cases and therefore miss cases carrying *MYC*-R. In this series, 4 of 7 false negative cases had high expression of MYC by IHC, suggesting that the combination of CD10, LMO2, and MYC may be useful to screen *MYC*-R. Likewise, MYC low expression may be useful to clarify false positive cases, as observed in 6 of 9 cases in the series. Nevertheless, the group of cases that we designated as dissociated CD10/LMO2 deserves further analyses to clarify their clinicopathological characteristics and weather combined with additional markers may help to identify *MYC*-R in aLBCL.

Finally, we wanted to know the approximate incidence of *MYC*-R among centers. Considering the characteristics of each center, we tried to clean external cases received in each center, assuming the hypothesis that incidences by centers should be similar. Then, before excluding cases outside the health area of influence of each, the incidences of *MYC*-R in aLBCL ranged from 0 to 26%. After exclusion, the incidence varied from 0 to 23%, with centers 1 to 3 showing a decrease of their incidence. These results may be related to the heterogeneity of aLBCL but raises the questions about how to approach to FISH testing and whether epidemiological differences exist among such health areas. To the best of our knowledge, studies evaluating the agreement of *MYC* interpretation by FISH concordance in aLBCL are lacking.

In conclusion, in this study we pretended to evaluate the clinical reproducibility of LMO2 immunohistochemical expression to screen *MYC*-R in aLBCL. In the first phase of the study, we observed high agreement between the observers interpreting LMO2, higher than the results obtained for MYC. In the second phase, we realized the variable approaches used to diagnose *MYC*-R, and we conclude that combining the profile CD10, LMO2, and MYC may be a useful method to screen the presence of *MYC*-R in aLBCL. As a result, all centers enrolled in the study included LMO2 in their diagnostic work up for aLBCL.

### Supplementary information

ESM 1 (DOCX 23.9 kb)

## Data Availability

The datasets generated during and/or analyzed during the current study are available from the corresponding author on reasonable request.
