# Supplementary material for: Round-robin testing for LMO2 and MYC as immunohistochemical markers to screen MYC rearrangements in aggressive large B-cell lymphoma
Source: Virchows Arch. 2023 Jun 27;485(2):307–14. doi: 10.1007/s00428-023-03584-9 (PMC11329383; doi:10.1007/s00428-023-03584-9)
Supplement: Supplementary file 1 — (DOCX 23.9 kb) [file 428_2023_3584_MOESM1_ESM.docx]

**Supplementary table 1.** Immunohistochemistry clones, FISH MYC probe and sources for the second phase, by laboratory

|  | **Center 1** | **Center 2** | **Center 3** | **Center 4** | **Center 5** |
| --- | --- | --- | --- | --- | --- |
| **CD10** | SP67  Ventana, Roche | 56C6  Novocastra, Leica | SP67  Ventana, Roche | SP67  Ventana, Roche | SP67  Ventana, Roche |
| **BCL6** | GI191E-A8  Cell Marque | GI191E-A8  Cell Marque | GI191E-A8  Cell Marque | GI191E-A8  Cell Marque | GI191E-A8  Cell Marque |
| **MUM-1** | GA644  Agilent, Dako | EP190  Cell Marque | MRQ-43  Ventana, Roche | EP190  Cell Marque | EP190  Cell Marque |
| **BCL2** | 124  Ventana Roche | 124  Ventana, Roche | 124  Ventana Roche | 124  Ventana Roche | 124  Ventana, Roche |
| **MYC** | Y69  Ventana Roche | Y69  Ventana, Roche | Y69  Ventana Roche | Y69  Ventana Roche | Y69  Ventana, Roche |
| **LMO2** | 1A9-1  Ventana Roche | 1A9-1  Ventana, Roche | 1A9-1  Ventana Roche | 1A9-1  Ventana Roche | 1A9-1  Ventana, Roche |
| **FISH probes*** | Agilent, Dako | Vysis, Abbott Molecular | Vysis, Abbott Molecular | Kreatech, Leica Biosystems | MetaSystems |
| **FISH interpreter** | Pathologist  (in-house) | Genetist  (in-house) | Genetist  (in-house) | Pathologist  (in-house) | Genetist  (external) |
| **Population covered (health area)** | 1.300.000 inhabitants | 800.000 inhabitants | 400.000  inhabitants | 400.000  inhabitants | 415.000  inhabitants |

* All centers used break-apart probes to detect *MYC*-R. Only centers 2 and 3 stained all rearranged cases with *MYC/IGH* dual-color dual-fusion probes. The source of these probes was Vysis, Abbott Molecular in both centers.

**Supplementary table 2.** Statistic Measures of LMO2 Protein expression in all cases and CD10 positive cases compared with the presence of *MYC* gene rearrangement by centers 1 to 4*

|  | **Center 1** | | **Center 2** | | **Center 3** | | **Center 4** | |
| --- | --- | --- | --- | --- | --- | --- | --- | --- |
|  | **All cases**  *N*=55 | **CD10+**  *N*=28 | **All cases**  *N*=35 | **CD10+**  *N*=15 | **All cases**  *N*=69 | **CD10+**  *N*=35 | **All cases**  *N*=36 | **CD10+**  *N*=13 |
| **Sensitivity** | 57% | 67% | 89% | 100% | 67% | 67% | 100% | 100% |
| **Specificity** | 77% | 94% | 42% | 56% | 67% | 85% | 71% | 92% |
| **PPV** | 44% | 80% | 33% | 60% | 23% | 60% | 17% | 50% |
| **NPV** | 81% | 79% | 100% | 100% | 93% | 88% | 100% | 100% |
| **Positive LR** | 2.47 | 11 | 1.54 | 2.25 | 2 | 4.33 | 3.4 | 12 |
| **Negative LR** | 0.55 | 0.35 | 0.26 | 0 | 0.50 | 0.39 | 0 | 0 |
| **Accuracy** | 69% | 82% | 54% | 73% | 67% | 80% | 72% | 92% |

*Center 5 not included since *MYC*-R were not detected; PPV: positive predictive value; NPV: negative predictive value; Positive LR: Positive likelihood ratio; Negative LR: Negative likelihood ratio

**Supplementary table 3.** Statistic Measures of LMO2 Protein expression in aLBCL including the Hans algorithm and MYC/BCL2 protein expression compared with the presence of *MYC* gene rearrangement

|  | **CD10+ cohort**  **LMO2**  *N*=101 | **GCB-like cases***  *N*=118 | **Non-GCB-like**  **cases***  *N*=88 | **GCB-like**  **DLBCL-NOS cases**  *N*=69 | **Non-GCB-like**  **DLBCL- NOS**  **Cases**  *N*=81 | **All cases**  **MYC/BCL2 protein***  *N*=198 |
| --- | --- | --- | --- | --- | --- | --- |
| **Sensitivity** | 75% | 68% | 67% | 67% | 100% | 56% |
| **Specificity** | 86% | 84% | 51% | 84% | 51% | 77% |
| **PPV** | 66% | 53% | 4,7% | 29% | 2,5% | 27% |
| **NPV** | 90% | 91% | 98% | 96% | 100% | 92% |
| **Positive LR** | 5.47 | 4.22 | 1.34 | 4.2 | 2 | 2.4 |
| **Negative LR** | 0.29 | 0.38 | 0.66 | 0.40 | 0 | 0.58 |
| **Accuracy** | 83% | 80% | 51% | 83% | 52% | 74% |

* Burkitt lymphoma was excluded from the analyzed cases
